# Association between the serum cotinine and trabecular bone score in the adult population: A cross-sectional study

**DOI:** 10.18332/tid/194680

**Published:** 2024-11-28

**Authors:** Shifu Bao, Weibu Jimu, Nai Mu, Fang Yan, Shuxing Xing, Zheng Zhou

**Affiliations:** 1 Department of Medicine and Life Sciences, Chengdu University of Traditional Chinese Medicine, Chengdu, China; 2 Department of Orthopedics, Chengdu Fifth People's Hospital, Chengdu, China; 3 Department of Geriatrics, Chengdu Fifth People's Hospital, Chengdu, China

**Keywords:** trabecular bone score, serum cotinine, cigarette smoke, NHANES, cross-sectional study

## Abstract

**INTRODUCTION:**

Trabecular bone score (TBS) is gaining attention as a novel approach for evaluating bone quality, as it provides insights into skeletal microarchitecture. We aimed to investigate the possible relationship between serum cotinine and TBS in the US population.

**METHODS:**

This cross-sectional study utilized data from the 2005–2008 National Health and Nutrition Examination Survey (NHANES). A total of 6961 adults aged ≥20 years with complete data on TBS and serum cotinine were included. Serum cotinine levels were measured using isotope-dilution high-performance liquid chromatography coupled with tandem mass spectrometry. TBS was derived from lumbar spine DXA images using the Med-Imap SA TBS Calculator. Weighted multivariable linear regression analyses were conducted, adjusting for age, sex, race, BMI, poverty income ratio (PIR), total spine bone mineral density (TSBMD), smoking status, C-reactive protein (CRP), total protein, blood urea nitrogen, serum creatinine, serum uric acid, serum calcium, alkaline phosphatase, and serum phosphorus. Subgroup analyses were stratified by sex, race, BMI, and PIR.

**RESULTS:**

A total of 6961 individuals were included in the analysis, with a mean (± SE) age of 45.20 ± 0.39 years, comprising 49.21% males and 50.79% females. The serum level of cotinine was negatively associated with TBS in the fully adjusted model. Specifically, for each unit increase in the log2-cotinine score, there was a corresponding 0.01 unit decrease in TBS (β= -0.01; 95% CI: -0.02 – -0.01, p=0.002). Participants in the highest tertile of serum cotinine had a significantly lower TBS compared to those in the lowest tertile (β= -0.01; 95% CI: -0.02 – -0.01, p=0.002). Subgroup analysis revealed a significant negative association between serum cotinine and TBS in females (β= -0.021; 95% CI: -0.03 – -0.01), but not in males. No significant associations were found when stratified by race, BMI, and PIR.

**CONCLUSIONS:**

Serum cotinine was negatively associated with TBS in US adults. Further large-scale prospective studies are still needed to explore the associative relationship of cotinine in TBS.

## INTRODUCTION

Osteoporosis is a prevalent systemic bone disease characterized by reduced bone mass and deteriorated microarchitecture, significantly increasing fracture risk, particularly in the elderly^1^. As the global population ages, the incidence of osteoporosis is rising, resulting in millions of fractures each year and imposing substantial economic burdens on healthcare systems^2^. While bone mineral density (BMD) is commonly used to diagnose and manage osteoporosis by assessing bone strength, it does not always correlate with fracture risk, as it may appear normal even when the risk remains high^3^. Therefore, BMD alone is insufficient for fully understanding bone health, particularly regarding bone microarchitecture. The trabecular bone score (TBS), derived from the analysis of dual-energy absorptiometry (DXA) images, provides a detailed assessment of bone microarchitecture, including trabecular distribution, connectivity, and structural integrity. TBS is calculated based on pixel variations in two-dimensional images, with dense trabecular structures exhibiting smaller and more numerous variations, while sparse structures show larger and fewer pixel differences^4^. Recent studies suggest that combining TBS with the Fracture Risk Assessment Tool (FRAX) enhances fracture risk prediction accuracy beyond that achieved with FRAX alone, offering a more comprehensive evaluation of fracture risk, especially in cases where BMD may not fully reflect bone strength^5^. Several studies have demonstrated a strong correlation between TBS and fracture risk, suggesting that TBS may be a more reliable predictor of fracture risk than BMD.

Smoking is a major risk factor for both osteoporosis and fractures^6^. Cotinine, a metabolite that remains in the body for an extended period, is recognized as a key biomarker for accurately determining tobacco smoke exposure^7^. Blood cotinine levels are more stable than those in urine, making serum cotinine a more dependable measure for evaluating tobacco exposure^8^. Cotinine not only reflects the level of tobacco exposure but is also linked to the negative effects of smoking on bone health, such as increased oxidative stress and the disruption of mesenchymal stem cell function, both critical for bone formation^9^. Despite the established link between smoking and bone damage, the connection between serum cotinine and TBS remains unclear.

The goal of this study is to examine the connection between serum cotinine levels and TBS in US participants, using data from the National Health and Nutrition Examination Survey (NHANES). Our hypothesis is that higher serum cotinine levels may be linked to lower TBS values.

## METHODS

**Figure 1:**
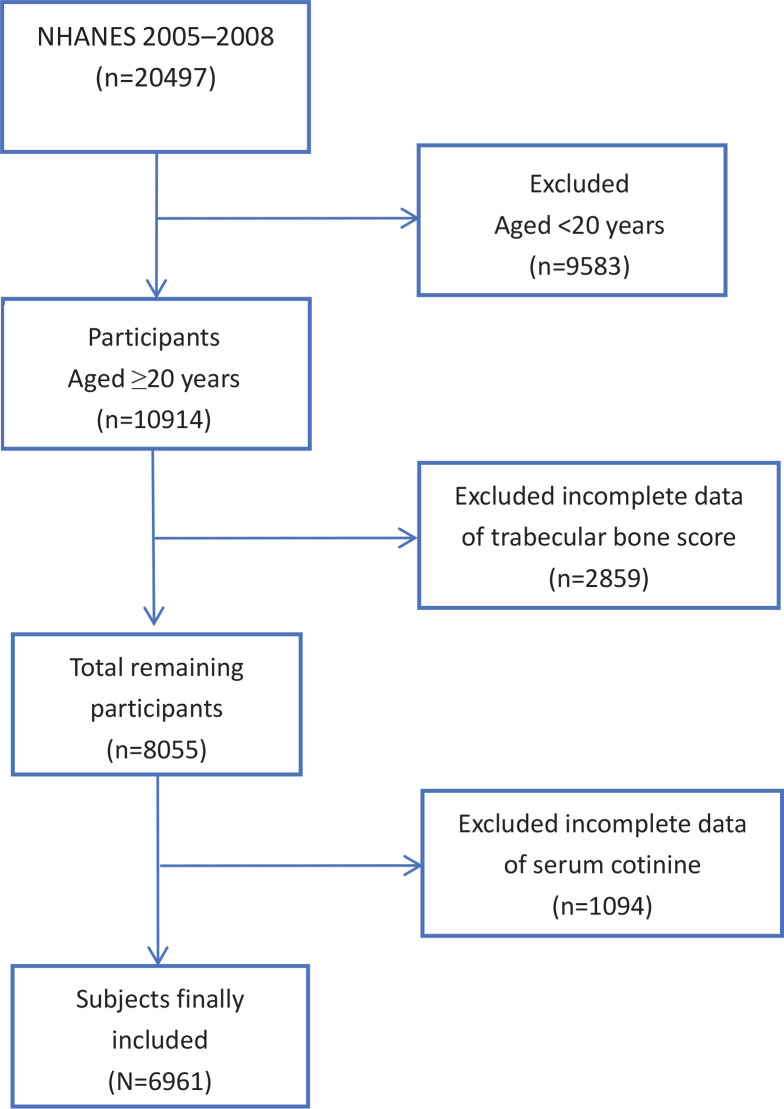
Flowchart for inclusion of participants, aged **≥**20 years, with complete data on TBS and serum cotinine, NHANES 2005–2008 (N=6961)

This research is a cross-sectional study utilizing data from the US NHANES database (http://www.cdc.gov/nchs/nhanes.htm), which reflects the health and nutritional status of the US population through a broad, representative survey^10^. The study protocol was reviewed and approved by the Ethics Review Board of the National Center for Health Statistics, with the most recent review in August 2022. All participants provided written informed consent. NHANES is a research program designed to assess the health and nutritional status of adults and children across different racial, ethnic, and socioeconomic groups in the United States. As TBS data were only available in the 2005–2008 NHANES cycles, the study included participants from the 2005–2006 and 2007–2008 periods. Initially, 20497 participants were considered, but after excluding those aged <20 years (n=9583), and those without TBS data (n=2859) or serum cotinine information (n=1094), the final analysis included 6961 individuals aged ≥20 years (Figure 1).

### Study variables

Blood samples from NHANES participants were drawn using venipuncture at mobile examination centers following standardized procedures. The serum cotinine concentration was determined using the isotope-dilution method coupled with high-performance liquid chromatography and atmospheric pressure chemical ionization tandem mass spectrometry (ID HPLC-APCI MS/MS).

TBS quantifies the microarchitectural integrity of trabecular bone in the lumbar spine by examining the variation in pixel intensity on lumbar spine DXA images. The TBS is derived using the Med-Imap SA TBS Calculator, which processes DXA data from vertebrae L1-L4 to produce a composite score for the lumbar spine. Devices like the Hologic QDR-4500A are commonly used for this evaluation, with the software utilizing an algorithm specifically designed to assess trabecular bone quality.

To guarantee data accuracy, the DXA scanning process adheres to rigorous protocols and standardized procedures, with comprehensive details available in the Body Composition Measurement Manual on the NHANES website (http://www.cdc.gov/nchs/nhanes.htm).

### Covariates

Building on prior research and clinical insights^11^, we accounted for covariates that could potentially impact the link between cotinine levels and TBS. The covariates considered in this study included age, sex, race, poverty income ratio (PIR), body mass index (BMI, kg/m^2^), total spine bone mineral density (TSBMD, kg/m^2^), smoke, C-reactive protein (CRP, mg/dL), total protein (g/dL), blood urea nitrogen (mg/dL), serum creatinine (mg/dL), serum uric acid (mg/dL), serum calcium (mg/dL), alkaline phosphatase (ALP, u/L) and serum phosphorus (mg/dL). PIR is a measure of family income relative to the federal poverty threshold, adjusted for family size and inflation, providing a standardized way to assess socioeconomic status^12^. BMI (kg/m^2^) is calculated as weight in kilograms divided by height in meters squared, and BMD refers to the amount of mineral content in a defined area of bone, expressed in kg/m^2^. Details of each variable were publicly available at www.cdc.gov/nchs/nhanes/.

### Statistical analysis

In line with Centers for Disease Control and Prevention (CDC) guidelines, statistical analyses were performed using the relevant NHANES sampling weights. The weights were adjusted according to the method outlined on the official NHANES website, using the weight variable *wtmec2yr* calculated as (1/4)×*wtmec2yr*. Continuous variables are expressed as means with standard error (SE), while categorical variables are represented as frequencies (n) and proportions (%). To assess differences among participants divided by cotinine tertiles, we applied either a weighted Student’s t-test for continuous data or a weighted chi-squared test for categorical data. In our preliminary analysis, the independent variables exhibited substantial skewness. To better meet the assumptions of linear regression, we applied a logarithmic transformation to the independent variables, thereby enhancing the robustness of the results. The association between serum cotinine and TBS was evaluated using multivariable linear regression across three different models. In Model 1, no covariates were adjusted. In Model 2, sex, age, and race were adjusted. Model 3 was adjusted as for Model 2 plus PIR, BMI, total spine BMD, CRP, total protein, blood urea nitrogen, serum creatinine, serum uric acid, serum calcium, ALP, serum phosphorus, and smoke. Stratification factors, including sex (male/female), race (Black/White/Other/Mexican American), BMI (<25; 25–29.9; >29.9 kg/m^2^), and PIR (≤1; 1.1–3.0; >3.0) were used for subgroup analysis of the correlation between serum cotinine and TBS^13,14^. These factors were also considered as pre-specified potential effect modifiers.

All data were processed and statistically analyzed using R 4.1.3. All statistical analyses were conducted using a significance level of p<0.05, with two-tailed tests applied throughout. The same significance threshold was maintained for interaction terms to ensure consistency with the primary analyses.

## RESULTS

### Baseline characteristics of participants

A total of 6961 participants were included, comprising 49.21% males and 50.79% females, with a mean (± SE) age of 45.20 ± 0.39 years. Cotinine levels for the three tertiles were categorized as follows: <0.027 ng/mL for tertile 1; 0.028–0.528 ng/mL for tertile 2; and ≥0.529 ng/mL for tertile 3. Significant differences were observed across the cotinine tertiles in terms of TBS, sex, race, PIR, BMI, serum uric acid, blood urea nitrogen, serum calcium, alkaline phosphatase, and serum phosphorus (all p<0.05). Table 1 presents the clinical and biochemical characteristics of the participants by cotinine tertiles.

**Table 1:** Characteristics of the study participants, aged ≥20 years, with complete data on TBS and serum cotinine, according to serum cotinine levels, NHANES 2005–2008 (N=6961)

*Characteristics*	*Total (N=6961) n (%)*	*T1 (N=2346) n (%)*	*T2 (N=2296) n (%)*	*T3 (N=2319) n (%)*	*p*
**Age** (years), mean ± SE	45.20 ± 0.39	48.67 ± 0.62	45.62 ± 0.69	41.24 ± 0.44	<0.0001
**Sex**					<0.0001
Female	3450 (50.79)	1397 (60.46)	1134 (50.69)	919 (41.04)	
Male	3511 (49.21)	949 (39.54)	1162 (49.31)	1400 (58.96)	
**Race**					<0.001
Black	1405 (10.16)	297 (6.48)	520 (11.43)	588 (12.66)	
Mexican-American	1370 (8.44)	617 (10.40)	440 (8.85)	313 (6.03)	
Other	838 (10.09)	325 (10.88)	295 (11.52)	218 (7.87)	
White	3348 (71.31)	1107 (72.24)	1041 (68.19)	1200 (73.44)	
**Smoke**					<0.0001
Yes	3307 (47.81)	630 (26.00)	759 (33.03)	1918 (84.65)	
No	3654 (52.19)	1716 (74.00)	1537 (66.97)	401 (15.35)	
	***Mean ± SE***	***Mean ± SE***	***Mean ± SE***	***Mean ± SE***	
**PIR**	3.13 ± 0.06	3.55 ± 0.07	3.12 ± 0.07	2.73 ± 0.06	<0.0001
**TBS**	1.39 ± 0.00	1.40 ± 0.00	1.38 ± 0.00	1.39 ± 0.00	0.003
**Total spine BMD** (gm/cm^2^)	1.04 ± 0.00	1.03 ± 0.00	1.04 ± 0.00	1.04 ± 0.00	0.08
**BMI** (kg/m^2^)	27.96 ± 0.12	27.82 ± 0.12	28.78 ± 0.20	27.30 ± 0.14	<0.0001
Alkaline phosphatase (mg/dL)	67.41 ± 0.46	65.70 ± 0.68	66.99 ± 0.60	69.56 ± 0.74	<0.0001
Serum creatinine (mg/dL)	0.90 ± 0.01	0.88 ± 0.01	0.90 ± 0.01	0.90 ± 0.01	0.24
Total protein (mg/dL)	7.12 ± 0.01	7.12 ± 0.02	7.14 ± 0.02	7.11 ± 0.02	0.19
Serum uric acid (mg/dL)	5.40 ± 0.03	5.22 ± 0.04	5.49 ± 0.03	5.51 ± 0.03	<0.0001
Blood urea nitrogen (mg/dL)	12.58 ± 0.11	13.24 ± 0.14	13.05 ± 0.16	11.44 ± 0.15	<0.0001
Serum phosphorus (mg/dL)	3.79 ± 0.01	3.81 ± 0.01	3.76 ± 0.01	3.80 ± 0.02	0.02
Serum calcium (mg/dL)	9.46 ± 0.02	9.46 ± 0.02	9.44 ± 0.02	9.48 ± 0.02	0.02
CRP (mg/dL)	0.38 ± 0.01	0.38 ± 0.02	0.37 ± 0.03	0.38 ± 0.02	0.95

Data are expressed as weighted means ± standard error (SE) or frequencies (n) and percentages (%). PIR: poverty income ratio. BMI: body mass index. BMD: bone mineral density. TBS: trabecular bone score. CRP: C-reactive protein. T1: cotinine level <0.027 ng/mL. T2: 0.028 ng/mL ≤ cotinine level ≤ 0.528 ng/mL. T3: cotinine level ≥ 0.529 ng/mL. P-values were calculated using weighted chi-squared tests for categorical variables and one-way analysis of variance (ANOVA) for continuous variables.

### The association between serum cotinine and trabecular bone score

The results of the multivariable regression analyses are presented in Table 2. Our findings indicate that higher cotinine levels are associated with a potential reduction in TBS. This association was significant both in Model 2 (β= -0.001; 95% CI: -0.002 – -0.001, p=0.0001) and Model 3 (β= -0.001; 95% CI: -0.002 – -0.001, p=0.001), suggesting that an increase in serum cotinine levels is linked to a decrease in TBS. Our results show that for each unit increase in the log2-cotinine score, there was a corresponding 0.01 unit decrease in TBS. Compared with the lowest cotinine tertile, participants in the highest cotinine tertile had significantly 0.01 decreased TBS than those in the lowest cotinine tertile (β= -0.01; 95% CI: -0.02 – -0.01, p=0.002).

**Table 2:** Association between the serum cotinine levels and the trabecular bone score of participants, aged ≥20 years, with complete data on TBS and serum cotinine, NHANES 2005–2008 (N=6961)

	*Model 1 β (95% CI), p*	*Model 2 β (95% CI), p*	*Model 3 β (95% CI), p*
**Continuous**			
Log2-transformed cotinine	0 (0.000–0.001), 0.096	-0.001 (-0.002 – -0.001), <0.0001	-0.001 (-0.002 – -0.001), <0.001
**Categories**			
Tertile 1 ®			
Tertile 2	-0.01 (-0.02–0.00), 0.03	-0.02 (-0.03 – -0.01), <0.0001	0 (-0.01–0.00), 0.26
Tertile 3	0.01 (0.00–0.01), 0.09	-0.02 (-0.03 – -0.02), <0.0001	-0.01 (-0.02 – -0.01), 0.002
p for trend	<0.001	0.86	0.03

Model 1: no covariates were adjusted. Model 2: adjusted for sex, race, and age. Model 3: adjusted as in Model 2 plus PIR, BMI, total spine BMD, smoke, CRP, total protein, blood urea nitrogen, serum creatinine, serum uric acid, serum calcium, serum alkaline phosphatase, serum phosphorus. For abbreviations see Table 1. ® Reference category.

Table 3 shows the multivariable regression analysis results examining the relationship between serum cotinine and the TSBMD. In Model 3, no significant association between cotinine and TSBMD was detected, regardless of whether cotinine was modeled as a continuous or categorical variable (all p>0.05). In conclusion, the results suggest a clear inverse relationship between higher cotinine levels and lower TBS, with this difference being notable.

**Table 3:** Association between the serum cotinine levels and the total spine bone mineral density of participants, aged ≥20 years, with complete data on TBS and serum cotinine, NHANES 2005–2008 (N=6961)

	*Model 1 β (95% CI), p*	*Model 2 β (95% CI), p*	*Model 3 β (95% CI), p*
**Continuous**			
Log2-transformed cotinine	0.0004 (-0.0003–0.0011), 0.2172	-0.0013 (-0.0021 – -0.0006), <0.0001	0.0006 (-0.0002–0.0014), 0.1395
**Categories**			
Tertile 1 ®			
Tertile 2	0.0095 (-0.0028–0.0219), 0.1239	-0.0025 (-0.0144–0.0094), 0.6740	-0.0024 (-0.0133–0.0086), 0.6448
Tertile 3	0.01 (0.0011–0.0189), 0.0284	-0.0157 (-0.0251 – -0.0064), 0.002	0.0051 (-0.0048–0.0150), 0.2810
p for trend	0.9279	0.0159	0.0817

Model 1: no covariates were adjusted. Model 2: adjusted for sex, race, and age. Model 3: adjusted as in Model 2 plus PIR, BMI, TBS, smoke, CRP, total protein, blood urea nitrogen, serum creatinine, serum uric acid, serum calcium, serum alkaline phosphatase, serum phosphorus. For abbreviations see Table 1. ® Reference category.

### Subgroup analysis

**Figure 2:**
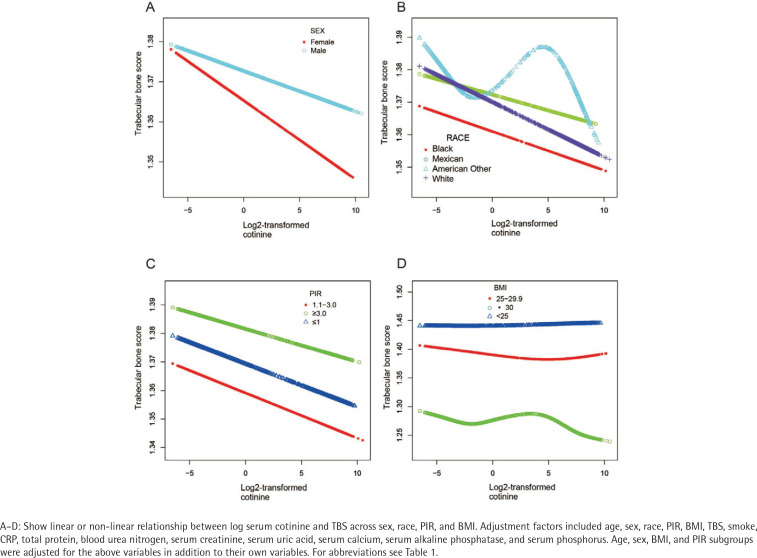
Association between log2-transformed cotinine and the prevalence of trabecular bone score (TBS) in different subgroups among participants, aged **≥**20 years, with complete data on TBS and serum cotinine, NHANES 2005–2008 (N=6961)

Table 4 presents a linear regression analysis of the specific relationship between serum cotinine levels and TBS. A significant association was found in females (β= -0.02; 95% CI: - 0.03 – -0.01). In the BMI-stratified subgroup, the interaction term had a p-value of 0.09. Although this did not meet the conventional significance threshold (p<0.05), it may still be considered marginally significant, indicating a potential interaction effect. Figures 2A–2D depict the associations between log-transformed serum cotinine and TBS across different populations, adjusting for sex, race, BMI, and PIR.

**Table 4:** Association between serum cotinine levels and trabecular bone score of participants, aged ≥20 years, with complete data on TBS and serum cotinine, stratified by sex, race, BMI, and PIR, NHANES 2005–2008 (N=6961)

	*Model 1 β (95% CI)*	*Model 2 β (95% CI)*	*Model 3 β (95% CI)*	*p for interaction*
**Stratified by sex**				0.017
Female	-0.008 (-0.019–0.002)	-0.035 (-0.046 – -0.024)	-0.021 (-0.033 – -0.008)	
Male	0.024 (0.011–0.038)	-0.008 (-0.019–0.003)	-0.009 (-0.022–0.005)	
**Stratified by race**				0.224
Black	0.008 (-0.016–0.033)	-0.007 (-0.028–0.014)	-0.003 (-0.024–0.018)	
Other	-0.01 (-0.039–0.020)	-0.031 (-0.060 – -0.002)	-0.024 (-0.057–0.009)	
Mexican American	0.002 (-0.019–0.022)	-0.015 (-0.034–0.004)	0.006 (-0.012–0.023)	
White	0.01 (-0.001–0.022)	-0.029 (-0.040 – -0.019)	-0.012 (-0.025–0.001)	
**Stratified by BMI**				0.09
<25	0.004 (-0.006–0.015)	-0.018 (-0.026 – -0.009)	-0.015 (-0.025 – -0.006)	
25–29.9	0.003 (-0.014–0.019)	-0.03 (-0.045 – -0.015)	-0.024 (-0.037 – -0.011)	
>29.9	-0.001 (-0.023–0.022)	-0.036 (-0.055 – -0.018)	-0.027 (-0.044 – -0.010)	
**Stratified by PIR**				0.425
≤1	0.022 (-0.006–0.051)	0.002 (-0.017–0.021)	-0.014 (-0.031–0.004)	
1.1–3.0	0.017 (0.003–0.031)	-0.021 (-0.035 – -0.006)	-0.016 (-0.030 – -0.002)	
>3.0	0.005 (-0.009–0.020)	-0.023 (-0.036 – - 0.010)	-0.008 (-0.023–0.008)	

Model 1: no covariates were adjusted. Model 2: adjusted for sex, race, and age. Model 3: adjusted as in Model 2 plus PIR, BMI, total spine BMD, smoke, CRP, total protein, blood urea nitrogen, serum creatinine, serum uric acid, serum calcium, serum alkaline phosphatase, serum phosphorus. For abbreviations see Table 1.

## DISCUSSION

This large-scale cross-sectional study, which analyzed combined data from the 2005 to 2008 NHANES, included a total of 36463 participants. We found that serum cotinine levels were independently associated with TBS. After adjusting for all covariates, our analysis showed a significant negative relationship between serum cotinine levels and TBS among US adults. Thus, we conclude that smoking cessation or reducing exposure to secondhand smoke may help improve TBS, enhance bone health, and lower the risk of fractures.

To the best of our knowledge, there is a limited amount of research exploring the connection between serum cotinine and TBS. TBS is gaining attention as a novel approach for evaluating bone quality, as it provides insights into skeletal microarchitecture. In contrast to BMD, which primarily quantifies bone mineral content, the TBS offers a detailed assessment of bone microarchitecture, specifically the quality and arrangement of trabeculae. This microarchitectural information allows TBS to detect more nuanced impairments in bone quality due to smoking. Some studies have highlighted TBS’s ability to differentiate between individuals with similar lumbar spine BMD (lsBMD) values, serving as a valuable complement to lsBMD. Furthermore, evidence suggests that integrating the FRAX with TBS can significantly enhance the precision of fracture risk prediction^15,16^. Research has shown that individuals who are actively or passively exposed to tobacco are at a higher risk of developing various health conditions, including reduced bone mass and an increased likelihood of osteoporotic fractures^17-19^. Cotinine is often chosen as the primary biomarker for assessing tobacco exposure because it tends to have higher concentration levels and a longer elimination half-life^20,21^. A cross-sectional study has suggested that reducing cigarette exposure and maintaining lower serum cotinine levels may promote bone health in adults, especially in women. This contrasts with the findings of our study regarding cotinine and BMD. A possible explanation lies in our use of TSBMD, as lsBMD data were unavailable in the NHANES 2007–2008; TSBMD spans multiple vertebral regions, potentially introducing regional variability that may have obscured the association between cotinine and BMD. Although many studies have demonstrated the connection between smoking and osteoporosis, the relationship between serum cotinine and TBS is still not well understood. In our study, we found a negative correlation between serum cotinine and TBS, which could be explained by several potential mechanisms.

Nicotine, the key addictive substance in cigarettes, interacts with nicotinic receptors found on osteoblasts^22^. Studies indicate that nicotine might impair the function of osteoblasts, thereby reducing bone matrix production, while also enhancing the activity of osteoclasts, which contributes to additional bone loss^23^. Nicotine is unstable, with the vast majority of absorbed nicotine rapidly metabolized into cotinine. While nicotine has a short half-life in the bloodstream (2–4 hours), cotinine has a longer half-life (16–20 hours), making it a more reliable indicator of long-term and sustained nicotine exposure, rather than the immediate effects of a single smoking event^7,21^. Smoking not only exerts a direct effect on bone health but also indirectly heightens the risk of osteoporosis through a variety of systemic mechanisms. Studies have shown that oxidative stress caused by smoking can trigger widespread inflammation, a chronic low-grade inflammatory state that is strongly linked to bone loss^24,25^. In smokers, the levels of inflammatory cytokines such as TNF-α and IL-6 are elevated, which stimulates osteoclast activity and accelerates bone resorption^26,27^. Vitamin D is essential for calcium absorption and bone mineralization, and its deficiency is directly linked to decreased bone density and the development of osteoporosis. Numerous studies have demonstrated that smokers tend to have lower serum vitamin D levels compared to non-smokers, suggesting that smoking may suppress the production of parathyroid hormone, calcifediol, and calcitriol^28,29^. Furthermore, research by Kassi et al.^30^ indicates that this deficiency is independent of age and sex, with smokers showing a 58% to 63% greater likelihood of vitamin D3 deficiency compared to non-smokers. Recently, there has been growing interest in the role of gut microbiota in osteoporosis, particularly regarding the composition of gut bacteria and its influence on bone health. Smoking has been found to disrupt gut microbiota balance and increase intestinal permeability, allowing harmful substances to enter the bloodstream and induce systemic inflammation^31,32^. This imbalance may lead to reduced nutrient absorption, which indirectly compromises bone health. In our study, when stratified by sex, the association was significant only among females (β= -0.02; 95% CI: -0.03 – -0.01, p<0.05). This result suggests that smoking may exert a stronger negative effect on bone health in women, possibly due to the regulatory influence of hormones like estrogen on bone metabolism. Smoking also affects hormone levels, particularly estrogen and testosterone. Testosterone plays a role in promoting osteoblast proliferation by binding to androgen receptors on these cells, while estrogen helps suppress osteoclast activity^33,34^. Although the effect of smoking on testosterone levels is still debated, its impact on women is clearer^35^. In women, cotinine can inhibit aromatase activity and enhance the hepatic breakdown of estradiol into 2-methoxyestrone, resulting in lower circulating free estrogen levels. This reduction leads to decreased osteoblast activity and proliferation, along with increased osteoclast activity and bone resorption^36^.

### Strengths and limitations

Our study has several strengths. First, we used a representative sample with a sufficiently large size. Second, we adjusted for potential confounding factors, ensuring more reliable results. Lastly, we performed subgroup analyses to explore the association between serum cotinine and TBS across different sex and ethnic groups.

Nevertheless, this study has several limitations. Firstly, the NHANES data, being cross-sectional, restrict our ability to establish a causal relationship between serum cotinine levels and TBS. Secondly, serum cotinine levels were measured only at a single point in time, which may not accurately capture long-term nicotine exposure. Additionally, reliance on self-reported data in NHANES introduces potential reporting bias that could influence the study’s outcomes. Lastly, given that the study sample primarily consists of individuals from the US, the applicability of these findings to populations in other regions may be limited.

## CONCLUSIONS

Our research identified an independent association between higher cotinine levels and lower TBS. This finding enhances our comprehension of the harmful impact of cigarette smoke on bone quality and underscores the critical role of smoking cessation in maintaining bone health. To substantiate these results, additional large-scale prospective studies are required.

## Data Availability

The datasets used in this study are available in online repositories. Details including the repository names and accession numbers are available from the following source: https://www.cdc.gov/nchs/nhanes/index.htm.
